# Chemical Recognition Cues in Ant-Aphid Mutualism: Differentiating, Sharing, and Modifying Cuticular Components

**DOI:** 10.1007/s10886-025-01562-w

**Published:** 2025-05-09

**Authors:** JESÚS FORONDA, LAURENCE BERVILLE, ESTEFANIA RODRÍGUEZ, ARÁNZAZU PEÑA, ELFIE PERDEREAU, MAR MONTORO, CHRISTOPHE LUCAS, FRANCISCA RUANO

**Affiliations:** 1 Institute for Agricultural and Fisheries Research and Training (IFAPA) La Mojonera, Almería, Spain; 2 Facultad de Ciencias, Universidad de Granada, Granada, 18071 Spain; 3 Instituto Andaluz de Ciencias de la Tierra (CSIC), Armilla, Granada, 18100 Spain; 4 Institut de Recherche sur la Biologie de l’Insecte (UMR7261), CNRS, University of Tours, Tours, France

**Keywords:** Chemical profiles, Cuticular hydrocarbons, Recognition, Mutualism, Formicidae, Aphididae

## Abstract

Aphid-tending ants form mutualistic associations with aphids. During their interactions, aphids and ants use both tactile stimuli and chemical cues to communicate. Recent studies suggest that ants modify the cuticular hydrocarbons of mutualistic aphids they attend, but it is unclear which compounds are implicated in recognition. Thus, we investigated the chemical basis for the discrimination between attended and unattended aphids, *Aphis gossypii* Glover (Hemiptera: Aphididae), by the ant *Tapinoma ibericum* (Santschi, 1925) including cuticular hydrocarbons (CHCs and non-CHCs) compounds in the analysis. Chemical profiles of 14 colonies of *A. gossypii* attended by ants for three days were significantly different from those of unattended aphids. These results show that contact with *T. ibericum* rapidly induces modification of the cuticular profiles of the aphids on which they feed. Moreover, the compounds of unattended aphid *A. gossypii* also change over time but differ from those of attended aphids. The main compound of the ant cuticle (3,15-di-MeC27), which is highly abundant in attended aphids, was identified as a possible recognition marker, but without forgetting other identified compounds that may also play a predominant role in the ant-aphid mutualistic interactions. These promising compounds represent opportunities for pest control strategies using chemical manipulations.

**Supplementary Information:**

The online version contains supplementary material available at 10.1007/s10886-025-01562-w.

## Introduction

Mutualism evolves and persists because the benefits of interactions between partners outweigh the costs. Ant–aphid interactions are a classic example of mutualism. Ants often care for aphids, protecting them from predators in exchange for honeydew, an excretion rich in carbohydrates, amino acids, and water, which is a reliable and valuable food resource (Pontin 1958; Way 1963; Skinner and Whittaker 1981; Sakata 1994). Ant-attended aphid colonies are therefore more stable and last longer (Dixon 1985). However, most of these interactions are relatively loose, and there is no strict relationship between a particular species of aphid and a particular species of ant (Stadler and Dixon 2005). Besides, several observations have shown that, even occasionally, ants also use the aphids they care for as a protein source (Pontin 1958; Sakata 1994). Typically, this results in ant visitation hierarchies, in which the ants favour the aphids that produce the best-quality honeydew (Addicott 1978; Völkl et al. 1999; Fischer and Shingleton 2001) and prey on the others (Sakata 1994, 1995; Offenberg 2001; Mooney and Tillberg 2005). Thus, to maximise their benefits, ants would have to be able to distinguish between individual aphids that provide abundant honeydew and those that provide less (Sakata 1995). Some authors have suggested that ants may mark aphids, depending on their capability to supply good honeydew, thanks to higher numbers of contacts (Sakata 1994). Thus, marked aphids are less likely to be predated by other ants (Sakata 1994; Endo and Itino 2012).

To communicate, aphids and ants rely on tactile and chemical stimuli (Kleinjan and Mittler 1975; Nault et al. 1976). Cuticular lipids—particularly cuticular hydrocarbons (CHCs) – are involved, among others, in species and colony recognition in insects (Howard and Blomquist 2005; Lucas et al. 2005; Blomquist and Bagnères 2010). CHCs and other non-CHCs compounds make up the insect cuticular lipid layer.

Due to their chemical properties, CHCs primarily act to limit insect water loss by creating a surface barrier (Howard and Blomquist 2005). The composition of CHCs varies greatly among species, but they mainly consist of a complex mixture of straight-chain saturated alkanes that can include one or several methyl groups (methyl alkanes) or present one or several double bonds (alkenes/alkynes) (Howard and Blomquist 2005; Martin and Drijfhout 2009). Depending on their structure, hydrocarbons can be grouped into different categories, and all possible combinations have led insects to develop very complex CHC profiles (Dahbi et al. 1996; Elmes et al. 2002). CHCs are used to distinguish colony members from others and therefore adopt friendly or aggressive behaviours towards them (Lang and Menzel 2011; Hojo et al. 2014; Hayashi et al. 2015). Mutualistic ants use CHCs to recognise aphids, their mutualistic partners (Lang and Menzel 2011; Hojo et al. 2014; Sakata et al. 2017; Endo and Itino 2012). Using cuticle extracts from aphids, it was proved that ants can discriminate between attended and non-attended individuals (Glinwood et al. 2003), suggesting that ants learn to associate aphid CHCs with honeydew rewards (Hayashi et al. 2015). According to another study (Lang and Menzel 2011), the n-alkane groups are a likely candidate because they are present in aphid CHCs and differ in relative abundance between mutualistic and non-mutualistic species. On the other hand, alkenes and methyl alkanes are favoured by most studies (Sturgis and Gordon 2012; Sakata et al. 2017). Conversely, in the presence of n-alkanes, ant aggressiveness was higher than towards entire aphid CHCs or methyl alkanes, implying that ants did not identify aphid dummies as partners based only on n-alkanes. The profile of the dummy CHCs excluded alkenes, as these compounds were not present in the studied aphids. Therefore, the roles of these compounds in aphid recognition could not be evaluated (Sakata et al. 2017).

Other compounds such as as fatty acids, alcohols, esters, aldehydes, and ketones form part of the insect cuticle and can protect insects against attack by microorganisms, parasites, and natural enemies (Gołebiowski and Stepnowski 2022; Michaud 2022). Thus, further identification of the whole compounds (CHCs and non-CHCs) used as partner recognition cues is required because there is currently no conclusive evidence as to which compounds act as recognition signals.

*Aphis gossypii* Glover (Hemiptera: Aphididae), known as the cotton aphid, is a cosmopolitan, polyphagous species that is widespread throughout the world (Kersting et al. 1999; Blackman and Eastop 2000), causing damage to various crops (Blackman and Eastop 2000). In the absence of natural enemies, an increased population growth rate has been observed when cotton aphids are protected by ants (Blackman and Eastop 2000; Rice and Eubanks 2013).

*Tapinoma* ants are part of the large Dolichoderinae subfamily (Ward et al. 2010). Cuticular hydrocarbons of several European *Tapinoma* species have been investigated previously, including *Tapinoma ibericum* Santschi 1925, which exhibits high diversity in CHC composition (Berville et al. 2013; Lenoir et al. 2023b). *Tapinoma ibericum*, granted as a species in 2017, is mostly found in Spain (Seifert et al. 2017, 2024). Out of its natural distribution range it has been considered an invasive species (Lenoir et al. 2023a, b) with the potential of forming large supercolonies (Seifert et al. 2017).

Understanding the chemical mechanisms underlying the interactions of ant-aphid mutualisms will help to better understand the role of semiochemicals in their evolution and regulation. Therefore, this study had two goals: we endeavoured to identify the cuticular profiles of two mutualistic species and how the aphid profiles are modified along time and during the mutualistic process. Accordingly, we analysed first the structural complexity of cuticular cues over a short period, and then we established the variation of aphid cuticular compounds in the presence of mutualistic ant species. The experiments were conducted with *T. ibericum* and *A. gossypii*, both of which occur naturally in greenhouses in Almeria, Spain. We hypothesised that ants’ cuticular profiles are transferred rapidly to the aphids they are nurturing.

## Materials and Methods

*Insect Collection and Rearing.* Samples of *T. ibericum* were collected from 14 different colonies located between greenhouses and distributed throughout Campo de Dalías (Almería), southeastern Spain (36°46’N, 2°41’W). In these areas, ants naturally attend to aphids and collect their honeydew. Sampling was carried out one week before the experiment, and approximately 500 workers per colony were collected. Individuals from different colonies were reared separately in round plastic containers (10 cm diameter and 5 cm high). The containers were covered with cardboard to keep the workers in the dark. Each container was connected by a vinyl tube (7 cm long, 0.4 cm in diameter) to another plastic container (25 cm x 15 cm x 7 cm) serving as the foraging area, whose edge was coated with Fluon (PTFE-30) to prevent ants from escaping. *Ephestia* sp. eggs (EPHEScontrol^®^ Agrobio SL) were provided as a protein source and water *ad libitum*.

*Aphis gossypii* colonies (*n* = 14) were collected from different crops in greenhouses located in the Campo de Dalías area. To allow colonies to develop and to ensure that there were no parasitoids, aphids were reared in separated culture chambers (27º C ± 1ºC, HR 60%, Photoperiod 16:8 h) in the facilities of the Sustainable Crop Protection department at the Institute for Agricultural and Fisheries Research and Training (IFAPA) La Mojonera for several generations (between 15 and 20 d, mean: 22.4 ± 4.8) on zucchini (*Cucurbita pepo* L.) from the variety Victoria^®^ (Clause, Spain). Using a single crop type for rearing aphids allowed to minimise CHC variations due to feeding. During the whole experiments, all zucchini plants were kept in separate entomological cylindrical cages (24 cm diameter, 38 cm height, Entomopraxis SCP Reference number G508) with screen aeration to conserve moisture, allowing to avoid the spread of individuals, natural enemies, and attraction of other aphid species.

*Experimental Design*. Manipulated individuals were always anaesthetised, using CO_2_ flow and placed in vials. Fourteen colonies of ants and aphids were used. For each colony, we considered one ant and five pooled aphids as a sample unit, with three replicates per colony. These three pseudo-replicates were later pooled during the statistical analyses. Two groups of aphids from 14 colonies were collected at T0 called “Aphid0”, before any contact between ants and aphids occurred (*n* = 14 colonies x 3 pools of 5 individuals per group, final sample size = 14). Then, two treatments were performed. Non-mutualistic aphids were raised for three days with no interaction with any ant, hereafter named “Aphid-” (*n* = 14). On the other hand, mutualistic aphids paired with an ant colony were collected after three days of mutualism, hereafter referred to as “Aphid+”. To do so, pots of zucchini containing a population of approximately 200 adult aphids were placed in the foraging area of an ant colony for three days, during which mutualisms were performed. Then, 15 aphids visually confirmed as attended by ants (Aphid+) of each colony and three ants actively performing mutualism (Ant+) were collected from each colony (*n* = 14 colonies x 3 individuals/pool, final sample size = 14). Note that ants at T0 were also collected for control purposes.

*Chemical Analyses.* A total of 252 samples were analysed. The first extraction was performed using 100 µl of heptane with 2.5 µl of 10^−5^ g/ml eicosane standard (219274, Sigma-Aldrich^®^). Then, the mixture was vortexed for 1 min, followed by a second extraction under the same conditions, resulting in a total volume of 200 µl. Samples were evaporated to dryness under gentle nitrogen flow. Then 10 µl of a solution containing 10^−5^ g/ml undecanoic acid methyl ester (methyl undecanoate U0250, Sigma-Aldrich^®^) in heptane was added. The full 10 µl of each sample was injected and analysed using a gas chromatography-mass spectrometer (GC-MS) (Agilent Technologies 7890B/7000 C; Les Ulis, France) coupled with MPS autosampler (Gerstel, RIC, Belgium). The GC-MS was equipped with an HP-5 capillary column (Agilent Technology, USA) of 30 m x 250 μm with a 0.25 μm stationary phase. Helium was used as the carrier gas at a constant flow rate of 2.3 ml/min. The temperature program of the column oven was from 40 °C to 320 °C at 5 °C/min with a hold time of 5 min at 320 °C. The electron impact was set to 70 eV. A CIS-4 injector (Gerstel) was set in solvent vent mode with the temperature ramping from 45 °C to 320 °C at a rate of 12 °C/s. The final temperature was maintained at 320 °C for 2 min. The septum purge flow was set at 3 ml/min with a purge flow to split vent of 60 ml/min at 2.57 min. The vent flow was set to 150 ml/min at 10 psi until 0.1 min. Mass spectra were acquired in full scan mode with a step size of 0.1 amu, a scan lapse of 250 ms, and a range of 40–550 amu.

Compounds were identified based on their mass spectra (Table 1), which were interpreted via fragmentation analyses and/or compared with spectra obtained by the National Institute of Standard and Technologies Library NIST MS Search 2.3 (NIST Mass spectral search program, 2017) and according to previous published data (Lenoir et al. 2023a). Both chromatograms and mass spectra were evaluated using MassHunter Qualitative Analysis B.10.00 (Agilent Technologies, Santa Clara, CA, USA). The individual abundance of each molecule was calculated using the height of quantifier ions by integrating the peaks over the extracted ion chromatograms (EICs, *m/z* ± 0.02) based on the chosen *m/z* values (qualifier ions) at a specific retention time using Agilent MassHunter Quantitative analysis for GC-MS (10.2, 2019). Over the 81 peaks analysed, seven comprised a mix of compounds with similar retention times (Table 1: P29, P35, P36, P43, P51, P54, P55). For these peaks, quantification was conducted using selected *m/z* values characteristic of the mixture. Peak areas were averaged per colony and each modality to obtain a single signal intensity value for each compound (*n* = 14). 

**Table 1.**
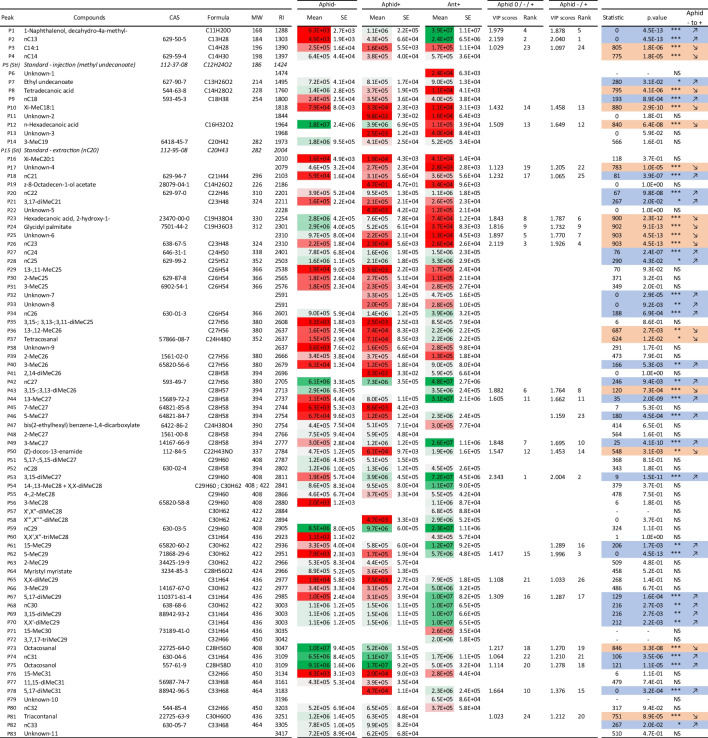
Abbreviation, name, CAS number (Chemical Abstracts Service), formula and molecular weight of the compounds found on *Tapinoma ibericum* and *Aphid gossypii* profiles for mutualistic ants (Ant+), Aphids at T0 (Aphid0), non-mutualistic aphids (Aphid-), and mutualistic aphids (Aphid+). RI: Kovats retention index. Mean area and standard error (SE) of each peak. The average area of a peak is represented by a color gradient (from green for the largest to red for the smallest). VIP scores and rank for aphids generated from the first component of the PLS-DA from Fig. 3 (comparison between Aphid 0/+/-) and from Fig. 4 (comparison between Aphid +/-). Statistics of the Kruskal-Wallis tests (Chi2 distribution) with p-values and the direction of the variation of the compounds from non-mutualistic aphids (Aphid-) to mutualistic aphids (Aphid+). Compounds growing to mutualistic aphids are blue colored and diminishing orange colored

*Statistical Analyses.* The area of each detectable peak in each chromatogram (EIC) was measured to assess and represent the overall distribution of the chemical profiles. Because some peaks contained more than one compound, the abundance of each molecule rather than peaks was used as units for statistical analyses. All statistical analyses and graphics were performed using R software (R 4.2.2). Data pre-processing consisted of first applying a correction based on the internal standard (methyl undecanoate: STD) to all samples. $\:Corrected\:{C}_{i}{S}_{i}area=\frac{{C}_{i}{S}_{i}\:area}{{~}^{{(S}_{i}\:STD\:area}\!\left/\:\!{~}_{Max\:STD\:area)}\right.}\:,$ with C_i_S_i_ area = Area of compound i in sample i; S_i_STD area = methyl undecanoate area of sample i; Max STD area = Maximum area of methyl undecanoate observed in all samples. As the resulting data matrix contained zeros (CHCs present in one profile but absent in another), an offset of 10% of the smallest non-zero value was added to all values to eliminate zeros. The final dataset was organised in a matrix with rows corresponding to the analysed samples and columns corresponding to the area of the compounds. Qualitative variables associated with the experimental design, such as species, colonies, time (T0 / T3), and mutualism (mutualistic / non-mutualistic), were also added to the matrix.

First, interspecific variability in profile composition was assessed using multivariate principal component analysis (PCA, ade4_1.7–22 and factoextra_1.0.7) based on the quadratic corrected area of the identified compounds. The contributions of variables (compounds) of the two main dimensions were calculated. A separate K-means cluster analysis (stats 4.2.2) was then carried out to group data points that had similar chemical profiles (Hartigan and Wong 1979). Each observation was allocated to the closest cluster, and the distance between each observation and the cluster was calculated from the Euclidean distance between the observation and the cluster center. The number of groups to be obtained is unknown. To select the optimum number, we used both the Elbow and average silhouette methods (factoextra_1.0.7). To explore the data and highlight clusters of individuals and compounds, a heat map was created (pheatmap_1.0.12).

To discriminate known groups of samples [Modalities: time (T0 / T3) or mutualism (mutualistic / non-mutualistic)], Partial Least Squares - Discriminant Analysis (PLS-DA) was performed. Data preprocessing consisted of averaging replicates for each sample (by colony and modality) to obtain a single signal intensity value for each compound. PLS-DA is well suited to discriminate groups based on chemical compounds that are more numerous than samples and that are multicollinear. The package mixOmics (6.22.0) (Rohart et al. 2017) was used to construct PLS-DA models. Samples were split into training and test sets. Because there were many more compounds than samples, the performance of the model was assessed before interpreting the score plots. This assessment was achieved by evaluating the number of samples that did not belong to the group predicted by the model. The classification error rate was computed using a double cross-validation scheme (Brereton and Lloyd 2014). The entire cross-validation procedure was repeated 10 times, resulting in 70 submodels for each experiment, the predictions of which were averaged. Permutation tests (999 permutations) based on the classification error rate were used to determine the significance of differences among groups (Westerhuis et al. 2008). To examine the relationships between groups, pairwise correlation tests based on cross-model validation were performed, and *p-*values were corrected for multiple testing using the False Discovery Rate (FDR) method (Benjamini and Hochberg 1995). The VIP scores summarise the contribution of a variable to the models (Eriksson et al. 2013). They were determined to capture the importance of each variable in the PLS-DA model using the ‘greater than one rule’ as criterion for variable selection (Eriksson et al. 2013). Finally, analyses were carried out to identify the compounds that evolved significantly with the mutualistic state between Aphid- and Aphid+. Because the data did not follow a normal distribution, a Kruskal-Wallis chi-square test and pairwise tests (Wilcoxon paired test) were performed.

## Results

*General Chemical Profiles of Ants and Aphids.* Ants *T. ibericum* and cotton aphid *A. gossypii* cuticles contained 81 peaks (and two standards) with 90 compounds with chain lengths ranging from 11 to 33 carbons (Table 1). These compounds consisted of 16 alkanes, 25 methyl-alkanes, 19 dimethyl-alkanes, two trimethyl-alkanes, one alkene, two methyl-alkenes, three aldehydes, four esters, one diester, one alcohol, one naphthalene, five fatty acids, and 11 additional compounds not identified yet. It is remarkable that most of the cuticular compounds in ants are CHCs, but in aphids the area of CHCs/non-CHCs peaks equal (Fig. S1, supplementary material). Most of the CHCs (> 75%) found in ants and aphids are therefore alkanes or methyl alkanes, while only ca. 4% are alkenes. Before mutualism, ants and aphids shared 57 peaks (70.3%). Ten peaks were present only in aphids (12%) and 14 peaks (17%) only in ants. In *T. ibericum*, four peaks accounted for 49.5% of the overall profile (P53: 3,15-di-MeC27; P42: nC27; P1: 1-Naphthalenol, decahydro-4a-methyl-; and P44: 13-MeC27), while 55 peaks accounted for less than 1% of the total. In *A. gossypii*, four peaks accounted for 50.4% of the overall profile (P75: Octacosanol; P74: nC31; P59: nC29; and P42: nC27), whereas 54 peaks represented less than 1%. Thus, the most abundant compound on aphid cuticles is not a CHC. In ants, lighter compounds were more abundant, whereas in aphids, the opposite occurred. The two largest peaks of *A. gossypii* (P75, P74) were ranked 26th and 40th, respectively, in *T. ibericum.* Moreover, only three molecules in the top ten were shared by ants and aphids (P42, P53 and P59), but in a different proportion, and among them, only nC27 (P42) emerged as dominant in both species. We include chromatograms of cuticular extracts of aphids (- and +) and ants in Fig. S7 (supplementary material).

**Fig. 1:**
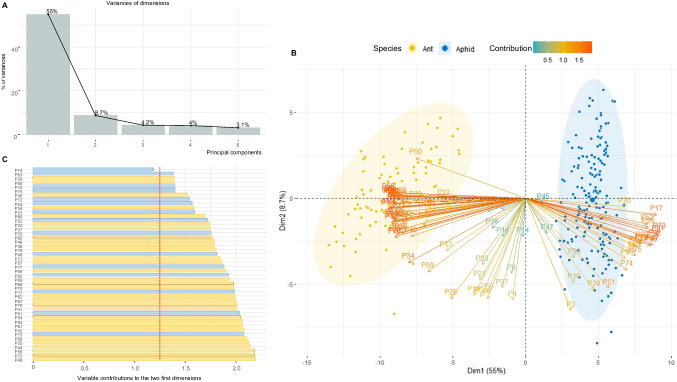
(**A**) Scree plot displaying how much variations of the data are captured by the first 5 principal components. Here, the top five axes capture 75% of variance. (**B**) Biplot of the Principal Components Analysis (PCA) based on the 252 samples, where it represents either ants (yellow) or aphids (blue), with 95% confidence ellipses. The axes show the principal component 1 and 2. The vectors are the loading vectors (compounds), whose components are colored depending on their magnitudes. A high cos2 (orange) indicates a good representation of the variable on the principal axes under consideration. In this case, the variable is positioned near the circumference of the correlation circle. (**C**) Bar plot of the main contributing variables (compounds) to the first two dimensions. The red dotted line indicates the expected average contribution. For a given component, the colors of the bar plot indicate if the contribution of the variables points toward ants (yellow) or aphids (blue)

*Cuticular differences between Ants and Aphids*. PCA was performed on the 81 peaks over the entire 252 samples to highlight general differences between ants and aphids. The PCA method is highly sensitive to outliers; thus, we searched for them using graphical checks. This resulted in no outliers being found. The amount of variation captured by each principal component from the data is shown in a scree plot (Fig. 1A). The PCA biplot explores the similarities among samples (Fig. 1B) based on the first two components by displaying both the PC scores of the samples (dots) and the loadings of variables (vectors). The variables are coloured according to their contribution to the principal components (gradient colours). The first and second axes

reveal a marked separation between ants and aphids (55% and 8.7% of the total variance, respectively). The main peaks contributing to PCA are represented in a bar chart (Fig. 1C). The red dotted line shows the expected uniform average contribution (1/number of variables = 1.23%). Therefore, variables with higher values than this threshold contributed more than the average. Of the 81 peaks, 45 contributed more than the average (P74 represented in Fig. 1C, is the first peak falling below the threshold). The colour of each bar represents whether the contribution of the peak is toward ants (orange) or aphids (blue).

**Fig. 2:**
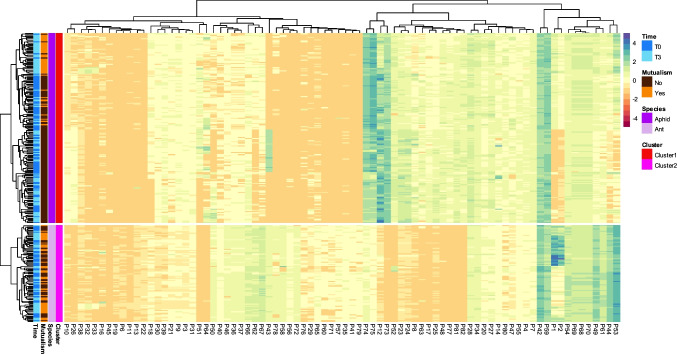
Heat map analysis of the abundances (peak area) of the chemical profiles of ants and aphids at different time intervals, and mutualistic conditions or not. Heat map represents unsupervised hierarchical clustering dendrogram (Euclidean distances) of groups (rows, *n* = 252). The rows display samples, and the columns represent the peaks (*n* = 81). The lower abundance of peaks in samples is displayed in dark brown, while higher abundance is displayed in dark green (the gradient is represented on the right). The annotation on the left side of the heatmap shows the distribution of either the modalities (species, mutualism state or time) or the clusters (1 or 2) calculated from the Euclidean distances

The optimal number of clusters obtained by Elbow and silhouette’s methods was found to be two. Thus, a separate K-means cluster analysis was performed in which all the ant and aphid samples were assigned to distinct groups. This clustering explained up to 68.4% of the total variance in our dataset. A heatmap (Fig. 2) was used to explore the data and highlight clusters, with rows representing several modalities (time, mutualism, species, clusters), and columns representing peaks. The heat map generates Cluster 1 (red) and Cluster 2 (pink) calculated using K-means that correspond perfectly to the species cluster [ants (light purple) *versus* aphids (dark purple)], but not to time [T0 (dark blue) *versus* T3 (light blue)], nor to mutualism modalities [No (brown) *versus* Yes (orange)]. We observed that certain peaks exhibited a significant disparity in their expression levels between the ant and aphid profiles. These profiles appear to be primarily characterised by some selected few peaks (including, but not limited to P12, P13, P44, P49, P53, P73, P74 and P75).

**Fig. 3:**
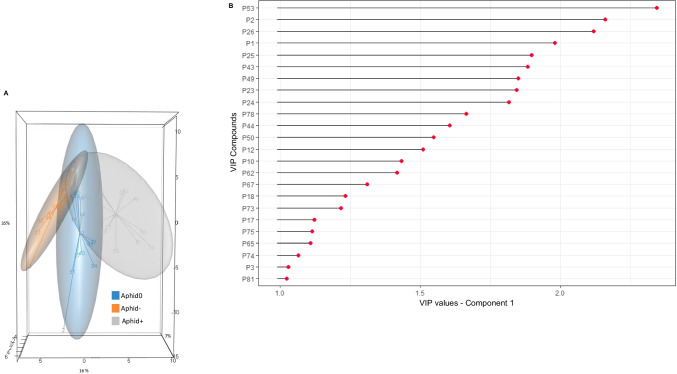
**(A)** Tri-dimensional partial least-squares discriminant analysis scatter plot based on the chemical profiles of Aphid0 (blue; Aphids at T0), Aphid+ (grey; mutualistic aphids), and Aphid- (orange; non-mutualistic aphids), with 95% confidence ellipses (Scores plot for Component 1: 35%, Component 2: 16%, Component 3: 7%). **(B)** Variable Importance in Projection (VIP, compounds with a VIP > 1 are deemed important) Scores, generated from the first component of the PLS-DA (Fig. 3.A), indicating the most discriminating compounds in descending order of importance

*Cuticular differences between Mutualistic and Non-mutualistic Aphids over Time.* To study the interaction between time and mutualism (Aphid0 *versus* Aphid- *versus* Aphid+) on the composition of the aphid profiles, we calculated the first PLS-DA on the 81 peaks (Fig. 3A). Model performance was assessed by evaluating the number of misclassifications (ncomp = 8). Cross-model validations (2CV), using both 6-fold (inner loop) and 7-fold (outer loop)

validation, yielded 30.9 ± 1.2% SE classification errors. Although we encountered a 30% error rate, this result supports the hypothesis of differences between the sampled groups (CER = 0.325; *P* = 0.001). Thus, all groups were pairwise compared (pairwise. MVA.test, FDR adjustment method), revealing significant differences (*P* = 0.002 for Aphid0 versus Aphid-, *P* = 0.0015 for both Aphid0 versus Aphid+, and Aphid- versus Aphid+). The results of the Variable Importance in Projection scores (= VIP scores), calculated for the PLS-DA to tentatively identify which features may discriminate between modalities (Fig. 3B), revealed that 24 peaks contributed the most to the separation of the three groups. The VIP scores and ranks of each variable in the first component of the PLS-DA are given in Table 1.

**Fig. 4:**
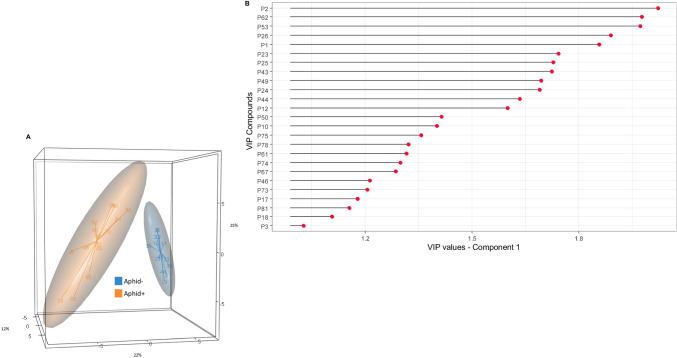
**(A)** Tri-dimensional partial least-squares discriminant analysis scatter plot based on the chemical profiles of Aphid+ (orange; mutualistic aphids) and Aphid- (blue; non-mutualistic aphids), with 95% confidence ellipses (Scores plot for Component 1: 25%, component 2: 22%, Component 3: 12%). **(B)** VIP scores generated from the first component of the PLS-DA (Fig. 4A), indicating the most discriminating compounds in descending order of importance

To study the impact of mutualism on the composition of aphid profiles (Aphid -/+), we calculated a second PLS-DA. Model performance was assessed and Aphid- and Aphid + were clearly separated from each other (CER = 0.0344; *P* = 0.001), with a mean risk of misclassification of 3.1 ± 0.95% SE. In the 3D score plots, the first three components explained 25%, 22%, and 12% of the variability (Fig. 4A). To summarise the contribution of the variable to the first component of the models, VIP scores and their rank were calculated (Table 1). Based on the first component, a VIP plot (Fig. 4B) was constructed, and 26 peaks were found to be significant for distinguishing Aphid- from Aphid+.

**Fig. 5:**
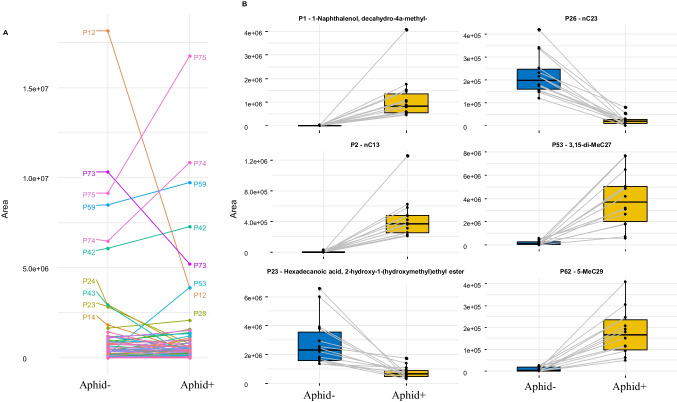
**(A)** The line plot illustrates the mean peak area from aphids’ profiles of the 81 compounds, from all the colonies for non-mutualistic aphids (Aphid-) and mutualistic aphids (Aphid+). The compounds listed are P75: Octacosanol, P74: nC31, P59: nC29, P42: nC27, P73: Octacosanal, P53: 3,15-di-MeC27, P12: n-Hexadecanoic acid, P28: nC25. **(B)** Box plots of the median and interquartile ranges of the six first VIP compounds from the second PLS-DA (Fig. 4). The gray lines connect the mean peak area from aphids’ profiles from the same colony for non-mutualistic aphids (Aphid- in blue) and mutualistic aphids (Aphid + in orange). The compounds listed are P1:1-Naphthalenol, decahydro-4a-methyl-, P2: nC13, P23: Hexadecanoic acid, 2-hydroxy-1-(hydroxymethyl)ethyl ester, P26: nC23, P53: 3,15-di-MeC27, P62: 5-MeC29

Finally, to identify which peaks were significantly different in aphids due to mutualism, we compared Aphid- and Aphid + profiles by performing *Kruskal-Wallis chi*^*2*^ tests (*KW chi*^2^ = 5725.2, df = 161, *P* < 2.2e^−16^) followed by Wilcoxon paired tests (Bonferroni corrected). The results are summarised in Table 1, showing whether the concentrations of the compounds significantly increased or decreased between Aphid- and Aphid+. These differences are represented in Fig. 5A, where each line illustrates the trend of the peak area (on average) due to mutualism. Among the most abundant compounds, five stand out as abundant, with two of them showing a significant decrease (P12 and P73) in attended aphids, while the other three exhibited significant increases (P53, P74, and P75). Interestingly, a few compounds appeared

to be significantly more abundant in Aphid- than in Aphid+ (highlighted in orange in Table 1). On the other hand, some compounds were not detected in Aphid- but were present in Aphid + and ants (Table 1).

The six first VIP compounds extracted from the second PLS-DA (Fig. 4) are represented in box plots in Fig. 5B with the median values and interquartile ranges for both treatments (P1: 1-Naphthalenol, decahydro-4a-methyl; P2: nC13; P23: Hexadecanoic acid, 2-hydroxy-1-(hydroxymethyl) ethyl ester; P26: nC23; P53: 3,15-di-MeC27; P62: 5-MeC29). Note that P53 (3,15-di-MeC27) is the major ant compound (18.8 ± 1.2% SE in average) and one of the most increased compounds in attended aphids (Fig. 5A, B; Table 2). Moreover, P53 was the first ranked compound in VIP analyses for the PLS-DA Aphid 0/-/+ (Fig. 3B) and the second ranked one (sharing the for the PLS-DA Aphid -/+ (Fig. 4B).

### CHCs Analyses

We run independent analyses considering only CHCs to test the cuticular differences between ants and aphids, and mutualistic and non-mutualistic aphids over time, as well as the heatmap clustering different treatments in our experiment. In this case, we obtained very similar results to those including also non-CHCs (Figs. S2, S3, S4, S5 and S6, supplementary materials).

## Discussion

The complex relationship between ants and aphids engenders profound alterations in the chemical compositions in the cuticle of attended aphids, thereby causing shifts in the abundance levels of numerous compounds. To comprehensively characterise and quantify these alterations, it was imperative to conduct a comparative analysis between profiles with and without mutualism. Through meticulous chemical analyses, discernible profiles emerged, characterised predominantly by alkane hydrocarbons, with nC27 (P42) being the most abundant and predominant compound shared by ants and aphids. Most identified compounds (> 75%) were alkanes or methyl alkanes, with alkenes constituting only around 4% of the total. This corroborates prior research findings (Sakata et al. 2017) and aligns with compounds previously identified in myrmecophilous and non-myrmecophilous aphid species. Nevertheless, one of our remarkable results is that CHCs showed highly abundant peaks on ant cuticles while in aphids, their abundance is nearly equal to that of non-CHCs, especially in the non-attended ones.

Before mutualism, a clear quantitative and qualitative demarcation was observed between the chemical signatures of the ants and aphids. Specifically, 10 peaks were exclusively

attributable to aphids (12%), while 14 peaks (17%) were uniquely associated with ants (Table 1). Moreover, among the 81 peaks analysed, 45 exhibited distinctive abundance ratios, effectively distinguishing between the two species. For instance, the first notable compounds included in this differentiation were P35, P44, P49, P56, P58, and P72 in ants and P17, P73, P81, and P82 in aphids, as shown in Fig. 1C. Two out of four of these notable compounds were non-CHCs, specifically Octacosanal (P73) and Triacontanal (P81). On the other hand, the results in Table 1 show that several peaks marked as notable compounds (P10, P12, P23, P24, P52, and P75) in Fig. 1C consistently demonstrate higher levels in aphids than in ants, encompassing the two unidentified peaks P17 and P25. Further examination of Table 1 revealed that few compounds present in aphids performing mutualism or not (Aphid- and Aphid+) were absent in ants performing mutualism (Ant+). Among these compounds, we could notice two aldehydes, P73 (octacosanal) and P81 (triacontanal), along with aliphatic or methylated alkanes like P82 (nC33), P77 (11,15-di-MeC31), P48 (2MeC27), and P63 (2-MeC29).

When ants attend aphids their chemical profiles undergo significant modifications, particularly affecting seven compounds. Among these, the abundance of n-Hexadecanoic acid (P12) markedly decreases in attended aphids. Additionally, compounds such as P74 (nC31) and P75 (octacosanol), which are relatively absent in ants, notably increase their presence in mutualistic aphids. In contrast, Peak 53 (3,15-di-MeC27), a major ant compound, exhibited a significant increase in abundance in attended aphids. When comparing the abundances of other contributing compounds (P10, P12, P17, P23, P24, P25) identified by PCA as principal components of aphids, we observed significantly higher concentrations in Aphid- compared to Aphid+. In this case, three of these important peaks correspond to non-CHCs: n-Hexadecanoic acid (P12), Hexadecanoic acid, 2-hydroxy-1- (P23) and Glycidyl Palmitate (P24).

To delve deeper into the potential impact of mutualistic interactions on the chemical profiles of aphids, we conducted a detailed analysis of the abundances of VIP compounds extracted from the PLS-DA analysis of Aphid 0/-/+ (see Fig. 3; Table 1). Notably, alkanes and methyl alkanes (such as P20, P27, P44, P49, P53, P78) exhibit higher abundances in ants than in aphids, suggesting the possibility that ants transfer these compounds as chemical marks during interactions. Other compounds involved are n-Aliphatic alcohols, which are considered feeding stimulants in some insect species (Mori 1982; Tibbets et al. 2008). Conversely, compounds like P25 (unknown-6), P73 (octacosanal), and P17 (unknown-4), either absent or present in low abundances in ants, experience a decrease in abundance in aphids upon the establishment of mutualism. Interestingly, hexacosanal (not present here) and octacosanal (P73), classified as long-chain aldehydes, have been reported to be repellent and cause toxicity in some insect species, apart from their putative role as alarm pheromones (Gade et al. 2016; Acheuk et al. 2017; Porras et al. 2022). Hexacosanal is not present in our current analyses, but two similar long-chain aldehydes were identified: octacosanal (P73) and triacontanal (P81). Moreover, these two compounds listed as VIP, are present only in aphids, attended or not. Their abundance decreases in Aphid + and both are absent in ants. All these compounds transferred or not transferred from ants, with increased or decreased abundances in aphids compared to the ants’ profiles, could be potential signals of mutualistic interactions.

Few compounds exhibiting statistical significance were not detected in Aphid- but were present in Aphid + and were found at higher abundance in ants. This is the case for unknowns P11, P13, P32, and P33, as well as the diMethyl compounds P58 (X, X-diMe C28) and P78 (5,17-diMeC31). These compounds are also promising candidates for transfer from ants to the cuticle of attended aphids. Additionally, other compounds are suitable for recognition marking (Fig. 4B), as three other VIP peaks experienced an increase in abundance when mutualism was established. Apart from P62 (5-MeC29), notable candidates include P2 (nC13), P1 (1-naphthalenol, decahydro-4a-methyl), and P53 (3,15-di-MeC27), which is the main constituent of the chemical profile of ants. This compound is nearly absent in Aphid0, with an average of 0.2% (± 0.1% SE) but represents 4.4% (± 0.5% SE) in Aphid+. In ants, di-Methyl alkanes are known to be involved in fertility signals to mark eggs (D’Ettorre et al. 2004) and appear to be determinants in colony recognition and in aggressive behaviours in ants (Astruc et al. 2001; Lucas et al. 2005; Sakata et al. 2017). Chemical marking of aphids by ants indicates that marked aphids are of particular importance to ants and should be carefully targeted for intensive mutualistic interactions.

Conversely, other compounds were present in Aphid- but were not detected in Aphid+. Among the peaks that experienced a significant decline in abundance, P12 (n-hexadecanoic acid) showed a decrease of more than one order of magnitude (Fig. 5A), as well as P23, a related acid (Fig. 5B). Hexadecanoic acid is commonly found in different insect orders, including many Aphididae (Thompson 1973), and it has been used as a soap acaricide for controlling soft-bodied pests (PPDB 2023). Therefore, if there is a decline in the abundance of both acids (P12 and P23), the chemicals that change after the onset of mutualism could be less prone to being washed or removed from the aphid’s cuticle due to the surfactant character of the fatty acids. Another group of compounds that showed decreased amounts after mutualism were aldehydes, including P73 (octacosanal), P37 (tetracosanal), and P81 (triacontanal). Although the role of aldehydes in insects is not fully understood, they might be used as alarm pheromones in response to threats (Bojke et al. 2020). A possible explanation could be that once mutualistic interactions have been established, ants would protect aphids against other enemies; thus, aphids would be less prone to release aldehydes to alert of immediate dangers. Another interesting compound was P43 (3,15-;3,13-diMeC26), abundant in Aphid- (peak area ca. 3.10^6^), but which disappeared after three days of mutualism. Such variations may be related to the physiological responses to mutualistic interactions. The behaviour of n-alkanes after mutualism establishment is not homogeneous. Indeed, the abundance of most n-alkanes (73%) increased, while for two of them, it remained unchanged (P52: nC28; P59: nC29), and the other two decreased (P4: nC14; P26: nC23). Interestingly, this last compound (P26) was the only VIP alkane whose abundance decreased in aphids after three days of mutualism. The exact role of saturated alkanes or other suitable candidates, in ant-aphid mutualistic communication should be determined through dedicated behavioural bioassays.

The chemical profiles of the aphids changed quickly within three days, independent of the existence of mutualism or not. This represents a very short period, highlighting the high plasticity of the chemical profiles of aphids, which could be particularly suitable for adaptation to various environments. The high variability of different situations can help individuals easily adapt to different environments. Several factors are known to influence the chemical composition of insect cuticles (Sprenger and Menzel 2020), but to our knowledge, the ability to change so rapidly in adult stages has not been previously emphasised. This rapid evolution of the cuticle chemical composition could be the basis of the practical adaptation of ant-aphids to mutualism. Nevertheless, the VIP compounds exhibited stability in the two PLS-DA analyses. Indeed, if we compare the top compounds listed as VIPs that discriminate between aphids over time (Aphid 0/-/+) and those studying the impact of mutualism (Aphid -/+) we find that nine out of the 10 top compounds from each modality coincide in composition. The two exceptions correspond to P62 (5-MeC29), which occupies the 3rd position in the discrimination of Aphid -/+, and P78 (5,17-diMeC31) which is in the 10th position for the Aphid 0/-/+ comparison. Therefore, both compounds could be important for mutualism interactions in insect communication.

This study highlights the substantial influence of mutualism on the chemical profiles of aphids, suggesting that mutualistic interactions with ants may play a pivotal role in modulating the production of specific substances within aphids, thus intricately shaping their chemical composition. Moreover, it promotes the need to broaden chemical analysis of insect cuticles to include compounds other than CHCs which may play significant roles in different insect species. Future research on ant-aphid mutualism should focus on exploring the responses of aphids over longer periods to evaluate whether these short-term changes in composition will last or will evolve to other scenarios. Moreover, future studies should focus on behavioural bioassays using candidate compounds detailed throughout this work, particularly compounds whose abundance significantly changes with the establishment of mutualism. This will allow us to identify active compounds that are most likely involved in mutualistic processes. These compounds represent opportunities for developing chemical manipulations for pest control treatments.

## Supplementary Information

Below is the link to the electronic supplementary material. 

ESM1 (PDF 708 KB)

## Data Availability

Availability of data and materials. On demand.
