# Supplementary material for: Chemical Recognition Cues in Ant-Aphid Mutualism: Differentiating, Sharing, and Modifying Cuticular Components
Source: J Chem Ecol. 2025 May 9;51(3):52. doi: 10.1007/s10886-025-01562-w (PMC12064600; doi:10.1007/s10886-025-01562-w)

Supplementary figures

Supplementary Figure 1

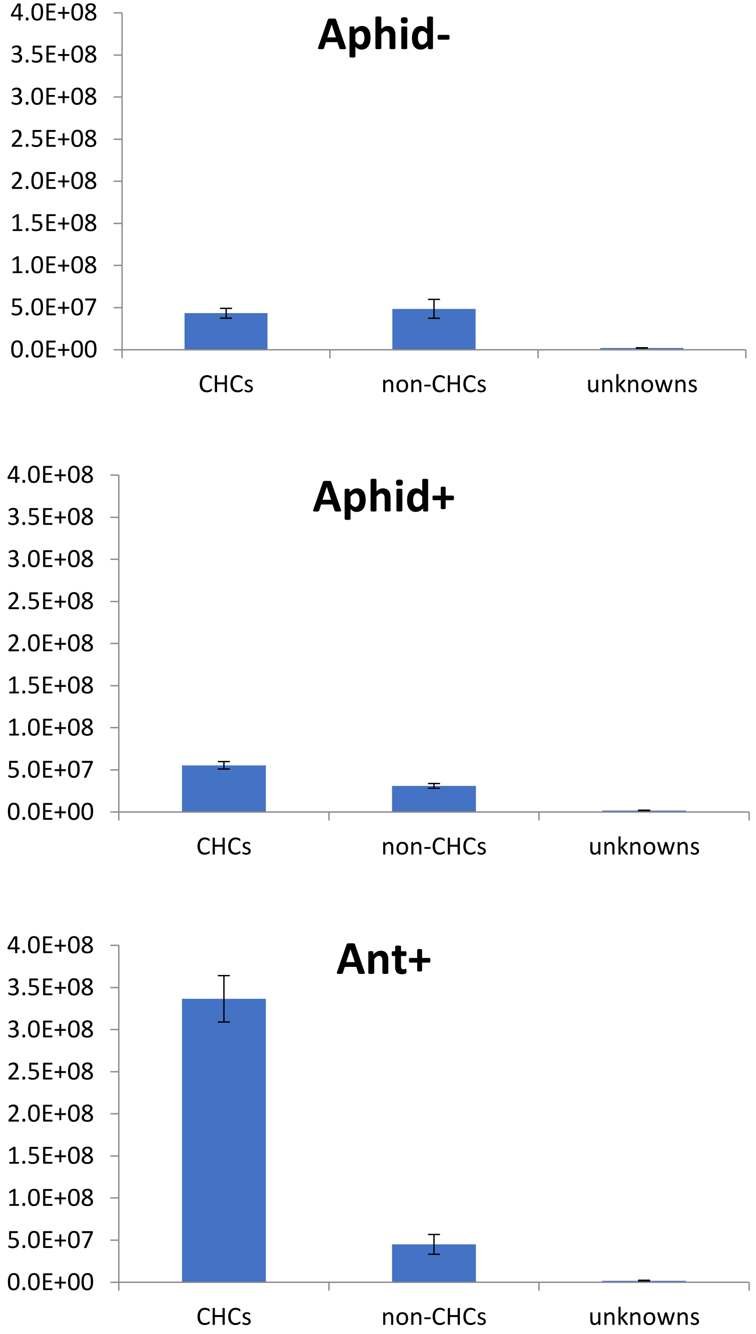

Supplementary Figure 2

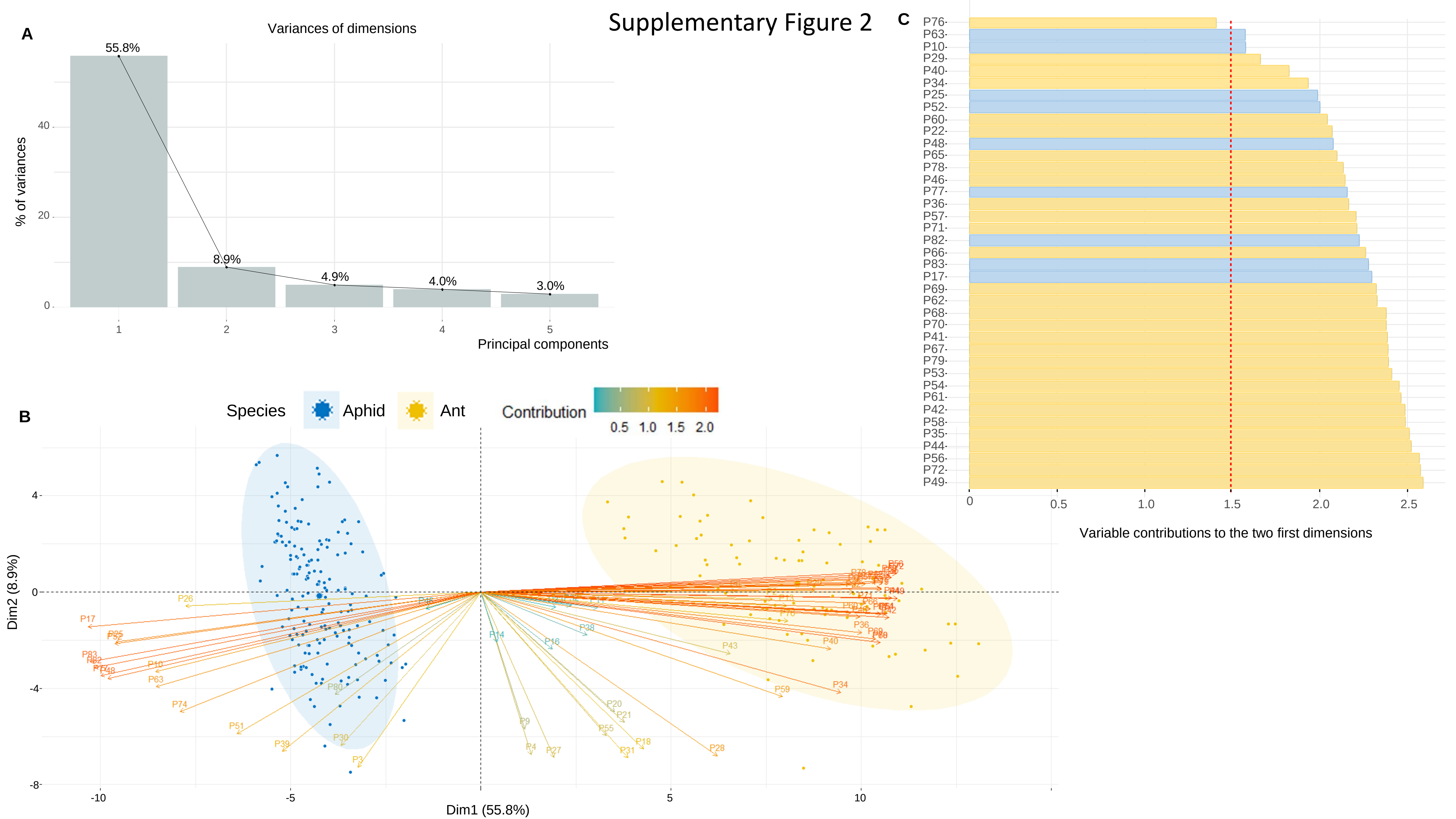

Supplementary Figure 3

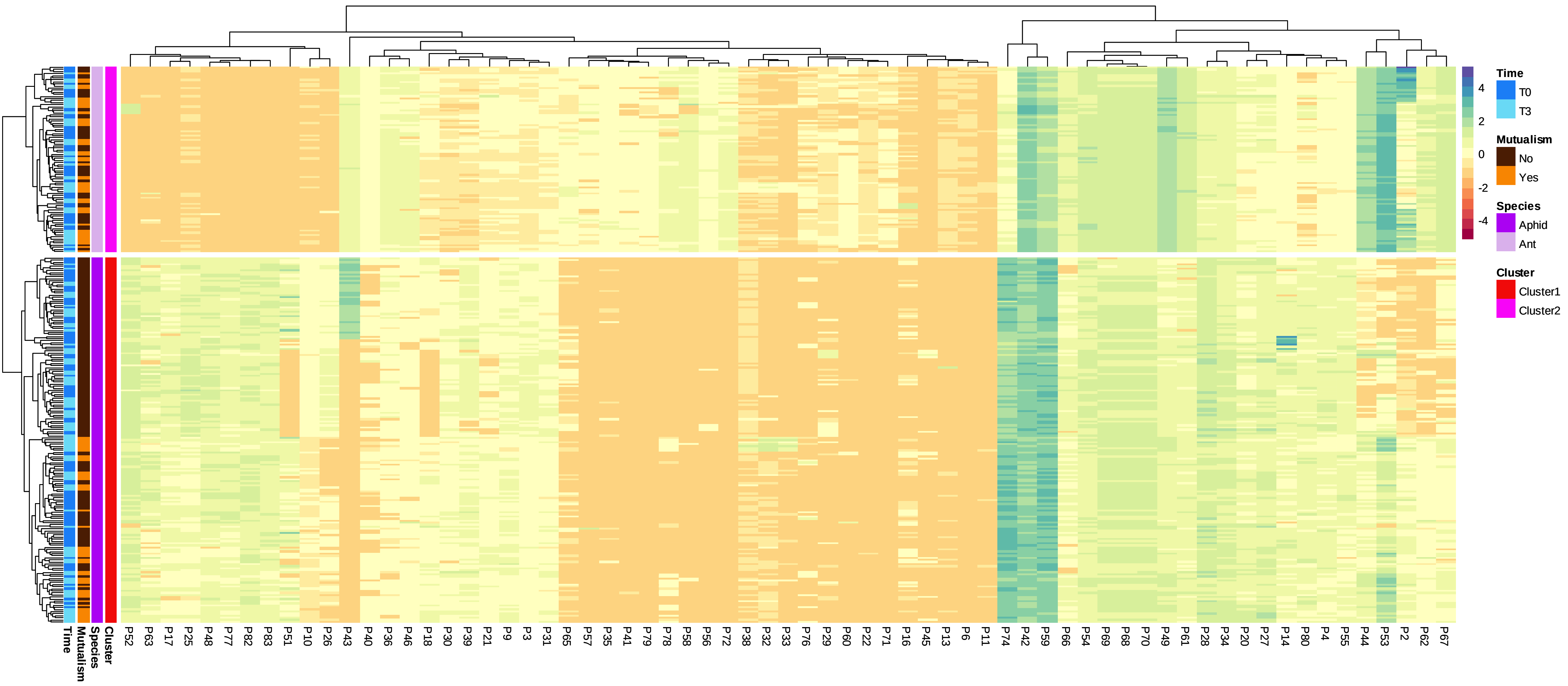

Supplementary Figure 4

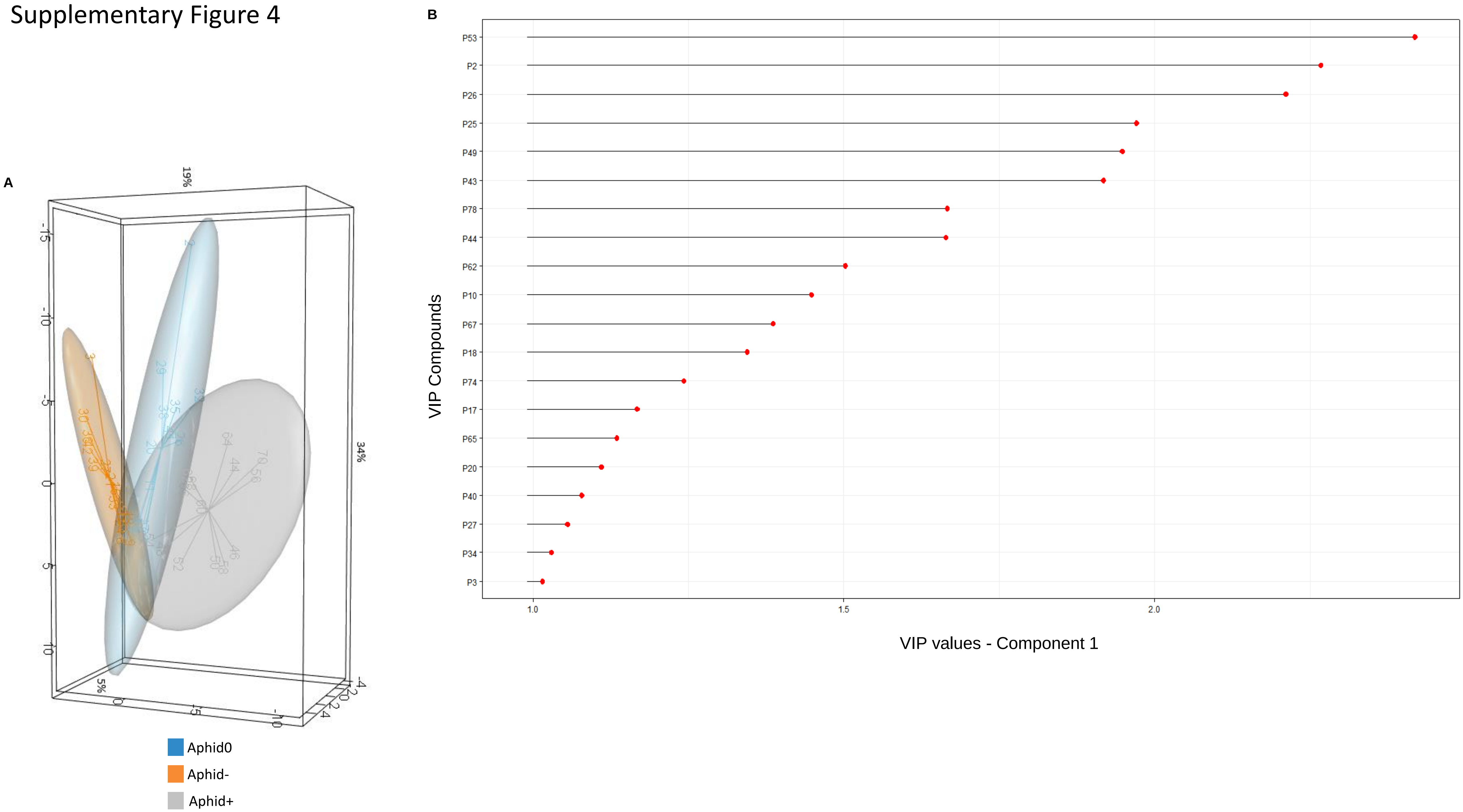

Supplementary Figure 5

A

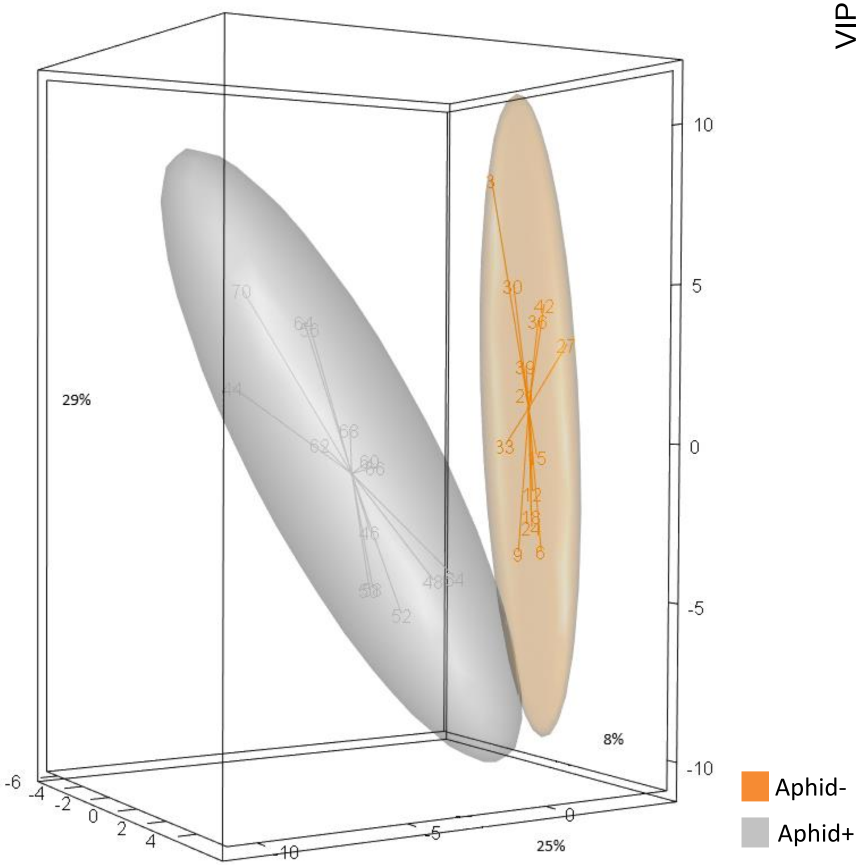

B

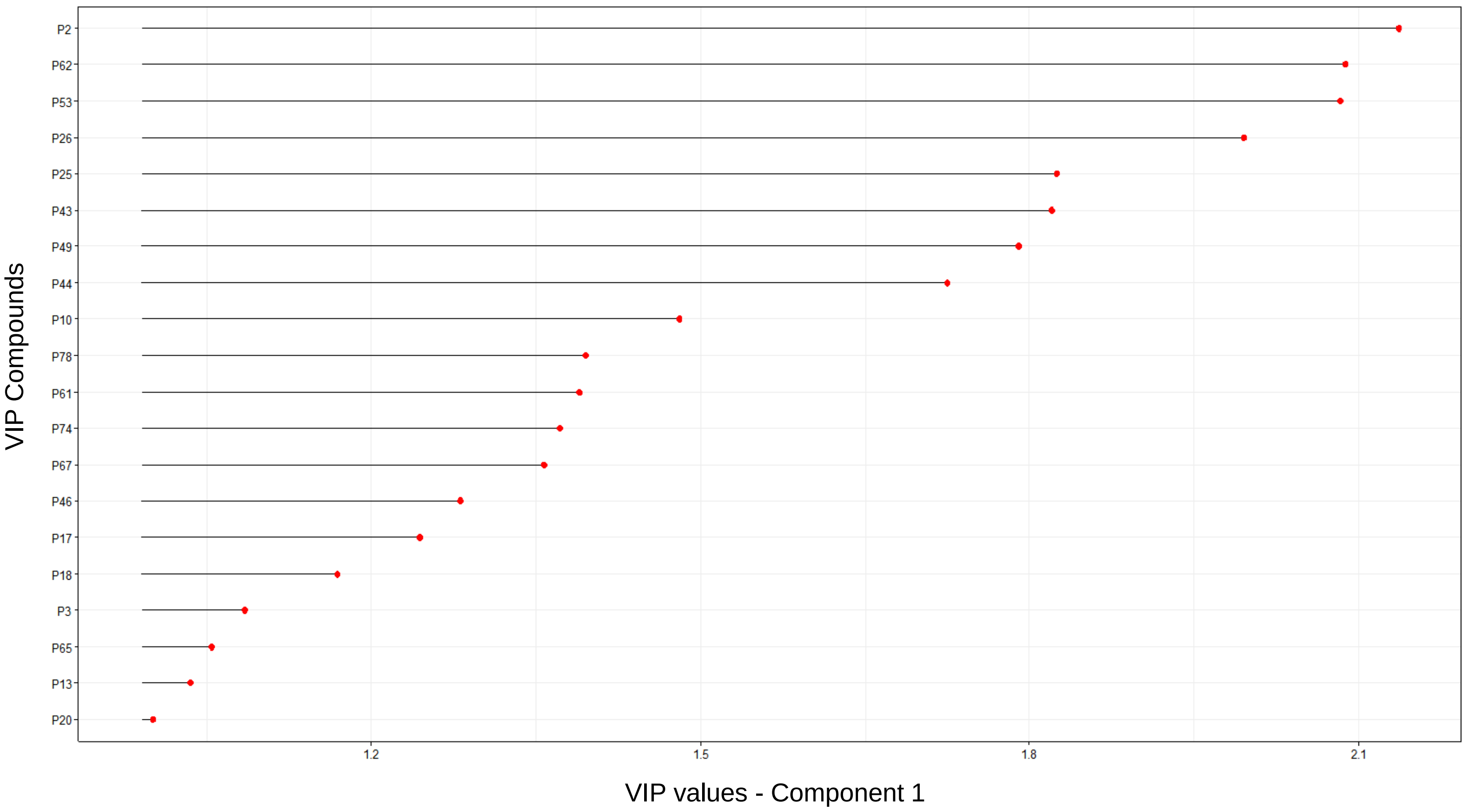

Supplementary Figure 6

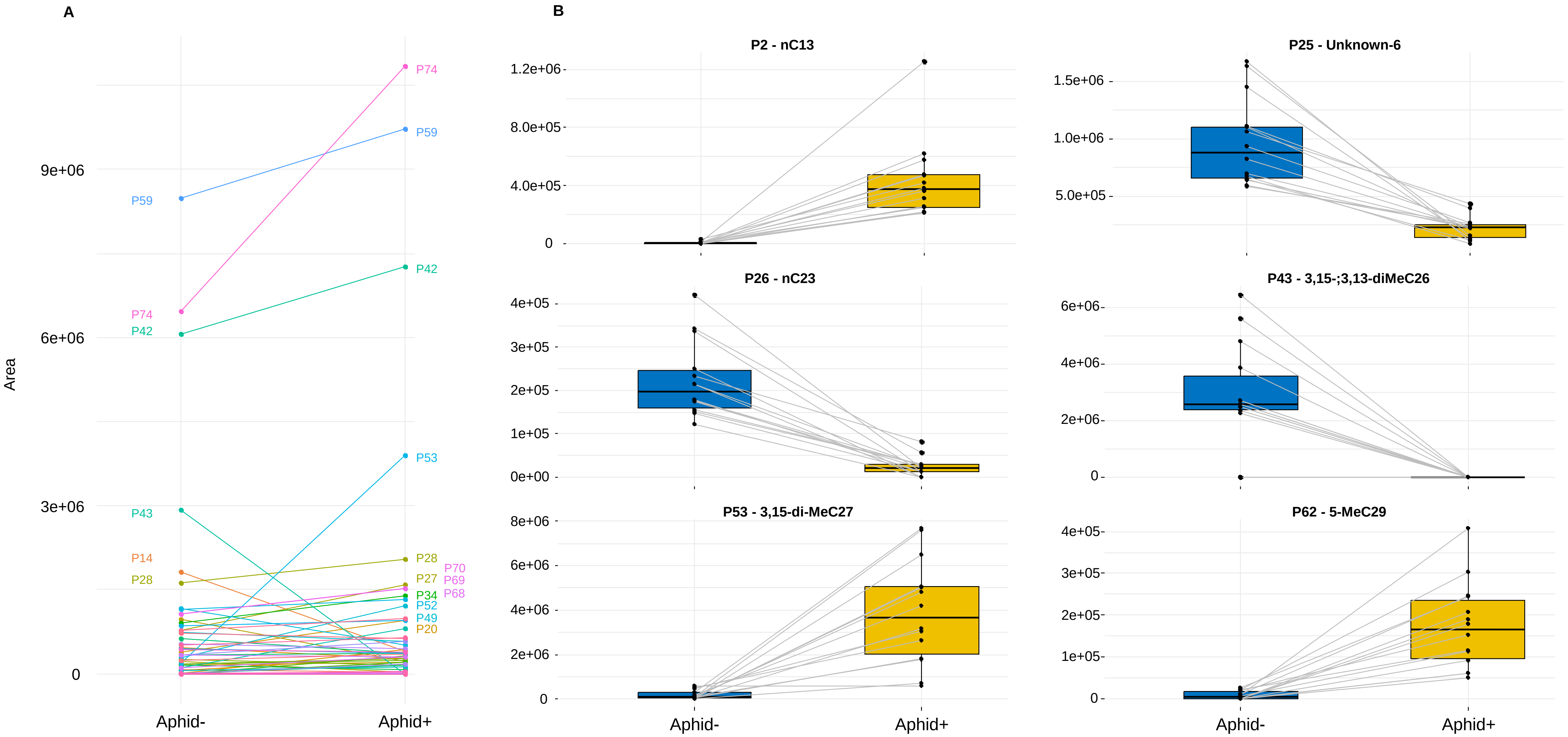

Supplementary Figure 7

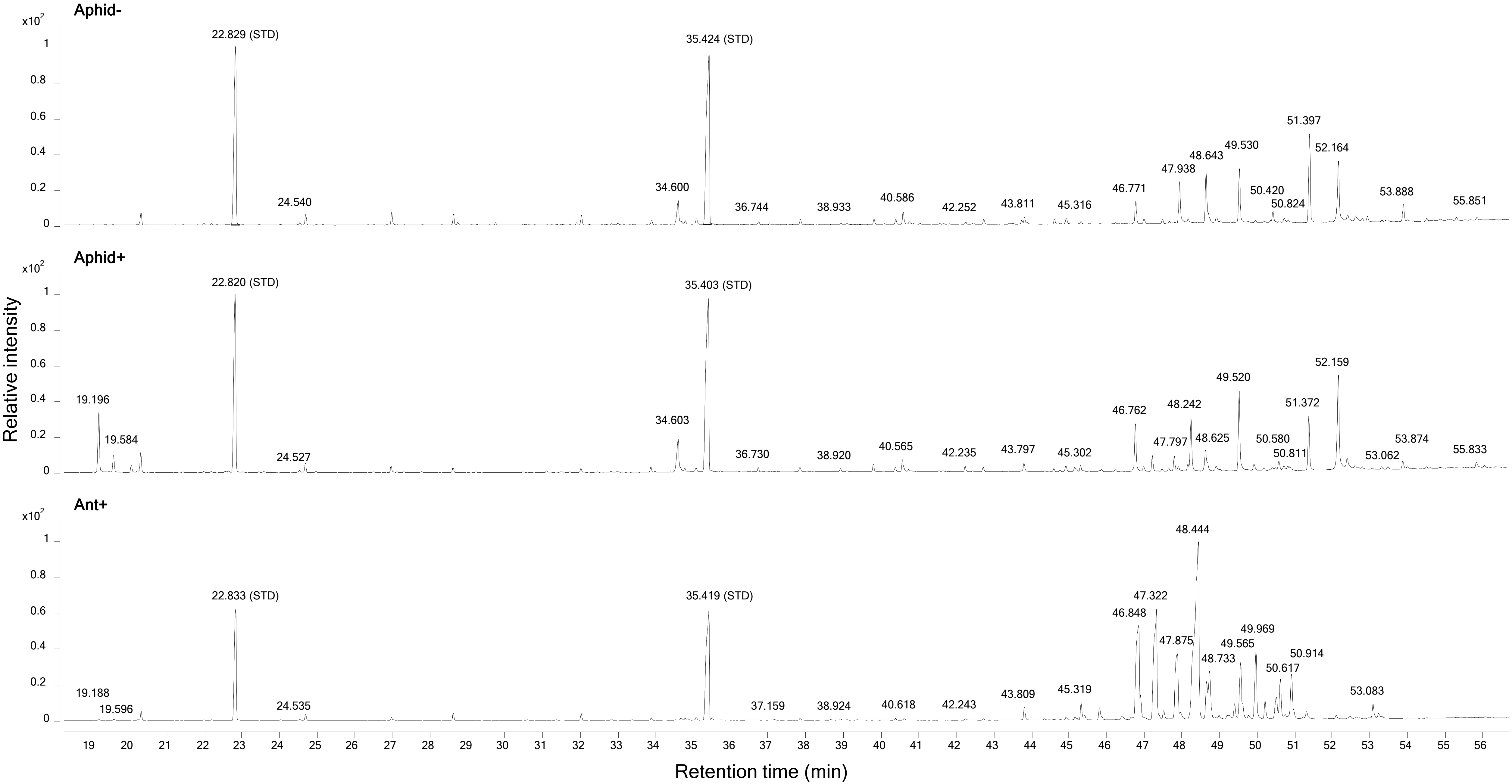

Supplement: Supplementary file 1 — (PDF 708 KB) [file 10886_2025_1562_MOESM1_ESM.pdf]
